# Leader-chasing behavior in negative artificial triggered lightning flashes

**DOI:** 10.1038/s41598-021-90940-x

**Published:** 2021-06-02

**Authors:** Fukun Wang, Jianguo Wang, Li Cai, Rui Su, Wenhan Ding, Zhiling Xu

**Affiliations:** School of Electrical Engineering and Automation, Wuhan University, Wuhan, 430000 China

**Keywords:** Atmospheric science, Electrical and electronic engineering, Natural hazards

## Abstract

Two special cases of dart leader propagation were observed by the high-speed camera in the leader/return stroke sequences of a classical triggered lightning flash and an altitude-triggered lightning flash, respectively. Different from most of the subsequent return strokes preceded by only one leader, the return stroke in each case was preceded by two leaders occurring successively and competing in the same channel, which herein is named leader-chasing behavior. In one case, the polarity of the latter leader was opposite to that of the former leader and these two combined together to form a new leader, which shared the same polarity with the former leader. In the other case, the latter leader shared the same polarity with the former leader and disappeared after catching up with the former leader. The propagation of the former leader in this case seems not to be significantly influenced by the existence of the latter leader.

## Introduction

During the propagation of leaders, there may exist competitions among different discharge processes. Some competitions will influence the final morphological structure of the leader channel. For example, Jiang et al*.*^1^ found that competition among clustered space leaders ahead of a stepped leader may determine whether the stepped leader produces branches and propagates in the original direction. Competition among different branches of a downward leader may determine the number and location of grounding points on a macro-scale^2–6^. And, competition among different induced upward connecting leaders may determine the number and location of grounding points on a micro-scale^7–13^.

Some competitions will not change the final morphological structure of the leader channel. Type *β*_2_ stepped leader, for instance, features a special propagation associated with the occurrence of one or more fast dart streamers^14–15^. These dart streamers travel rapidly down along the previously formed channel and cease when they have caught up with the leader tip. The dart streamer and type *β*_2_ stepped leader compete in the same channel, and therefore, this competition will not directly cause morphological change.

Schonland^14^ classified negative stepped leaders into two categories: *α* type and *β* type. The characteristic of type *β* leaders is the discontinuity in their downward development. Type *β* leader has longer and brighter steps with a high propagation speed near the cloud base. When type *β* leader approaches the ground, it decreases in speed and brightness and has shorter steps. The subtypes *β*_1_ and *β*_2_ were made by Schonland^14^; Type *β*_2_ leader is a relatively rare variant of type *β* leader whose propagation is associated with the occurrence of dart streamers. The researches on type *β*_2_ stepped leaders are rare. Campos et al*.*^15^ made detailed research on seven cases of type *β*_2_ stepped leaders and proposed that dart streamer is the visible manifestation of one or more recoil leaders. These recoil leaders begin inside the cloud and connect to the in-cloud, positive portion of the bipolar, bidirectional leader, and then travel downward to the lower end of the negative stepped leader path.

In this paper, we reported two cases of leader-chasing behavior, which are observed for the first time, during the propagation of dart leaders in two artificial triggered lightning flashes. Each of the observed cases contained two leaders occurring one after another, before the same return stroke. The two leaders propagated in the same channel and the latter (named chasing leader) caught up with the former (named chased leader) before reaching the ground. After they met each other, only one leader (named united leader) remained in the recorded frames, which initiated a return stroke later. The leader-chasing behavior here and the type *β*_2_ stepped leader reported earlier involve a similar propagation process on optical records, i.e. two leaders propagate and compete in the same channel. Different from type *β*_2_ stepped leaders, the leaders reported here are dart leaders descending continuously with a higher speed in the residual channel left by preceding return strokes instead of the virgin air, whose optical characteristics have been well studied^16–20^.

## Analysis and results

### Case I

**Figure 1:**
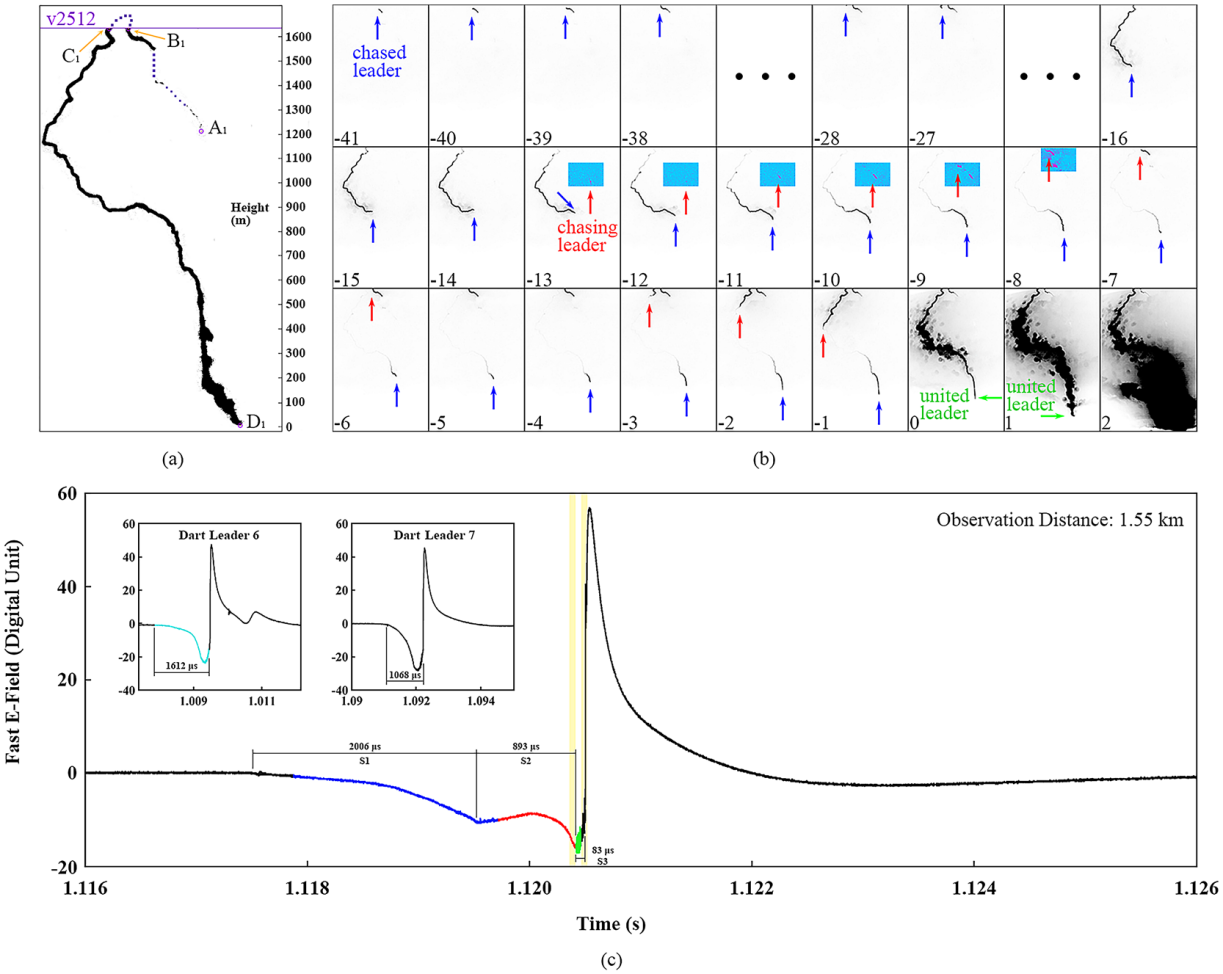
Optical recordings and corresponding electric field signatures of the leader-chasing behavior in Case I. (**a**) A processed frame showing the path for the leaders. The purple line indicates the upper boundary of the high-speed camera’s FOV, and the purple dotted line is the speculative channel structure. (**b**) A sequence of cropped high-speed frames showing the leader-chasing behavior, with background removed, intensity inverted, and contrast enhanced. The arrows with different colors indicate the position of each leader’s head. Some areas in Frames − 13 to − 7 were colored with contrast further enhanced. (**c**) Corresponding electric field signature with two insets showing counterparts.

Case I was an altitude-triggered lightning flash with eight return strokes, whose leader-chasing behavior occurred before the 8th return stroke. Figure 1 illustrates the high-speed frames and corresponding electric field signatures of the leader-chasing behavior. Figure 1a shows the path of leaders with some nodes marked out. The purple line indicates the upper boundary of the field of the view (FOV) of the high-speed camera and the dotted line reflects the portion of the channel which was out of the FOV or hidden by the cloud. Figure 1b illustrates a sequence of cropped high-speed frames showing the leader-chasing behavior, with background removed, intensity inverted, and contrast enhanced. The head of the chased leader I reached the boundary (about 1600 m above the ground) of the FOV in Frame − 38 and then propagated outside. In Frame − 28, the head of the chased leader I entered the FOV again and propagated towards the ground. In Frame − 13, the chasing leader I began its quite faint initial propagation as shown in the rectangle portion. The chased leader I had a similar faint initial propagation which is not shown here. From Frames − 13 to − 5, during the occurrence and development of the chasing leader I, the propagation of the chased leader I was weaker and weaker as indicated by its gradually shortened luminous channel. The tail of the chased leader I began to extend backward from the Frame − 4, making the luminous channel longer. At the same time, the chasing leader I propagated at a higher speed and soon caught up with the chased leader I. The height at which these two leaders met in Frame 0 was between 800 and 1200 m, and the united leader I initiated the return stroke in Frame 2.

Figure 1c shows the electric field signature of this process, with two insets showing those of two dart leaders which occurred within about 100 ms before the leader-chasing behavior. The two dart leaders (Darts leader 6 and 7) initiated the 6th and 7th return stroke, respectively. We color the section corresponding to Frames − 41 to − 14 blue, Frames − 13 to 0 red, and Frame 1 green. The specific sections corresponding to Frames 0 and 2, where the two leaders met and the return stroke occurred, are marked out with two yellow shadows. We divide the electric field signature before the 8th return stroke into three segments: S1, S2, and S3. A distinct difference between the signature variation before the 8th return stroke and Dart leaders 6 and 7 is the segment S2, where the electric field turns to increase gradually, followed by a fast decrease afterwards. Considering that the high-speed frames may fail to record the initiation of the chasing leader I, the electric field variation of segment S2 is directly related to the propagation of the chasing leader I. The total duration of S1, S2 and S3 is 2982 μs, which is much longer than those of Dart leaders 6 and 7.

**Figure 2:**
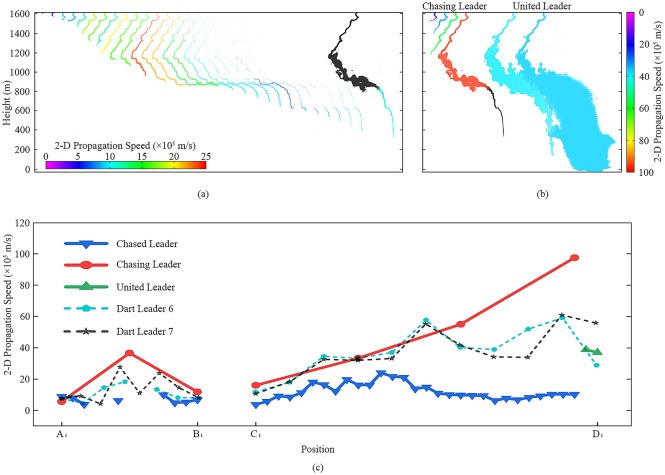
Propagation speeds of leaders in Case I. (**a**) The speed-position figure of the chased leader I. (**b**) The speed-position figures of the chasing leader I and the united leader I. (**c**) The propagation speeds variation of the chased leader I, the chasing leader I, and the united leader I, with two dart leaders as counterparts.

Figure 2 illustrates the propagation speeds of leaders in Case I. In Fig. 2a,b, we pick out and color the luminous channels of the chased leader I, chasing leader I, and united leader I from each frame shown in Fig. 1b. The color of the specific channel here reflects the propagation speed calculated on the position of the leader’s head. It is worth noting that the relationship between color and speed in the two figures is different. Figure 2a,b can tell us the speeds of these leaders when they propagated to a specific position. The tail of the last channel in Fig. 2a is black, because the two leaders met in the corresponding frame, and we assume the black part belongs to the chasing leader I instead of the chased leader I. The first channel in Fig. 2b is black for the similar reason. It can be seen from Fig. 2a that the propagation speed of the chased leader I increased first and then decreased. The speed of the chased leader I’s head decreased with the decrease of the luminous channel length.

Figure 2c illustrates the propagation speeds of the chased leader I, chasing leader I, and united leader I, with two dart leaders mentioned before as counterparts. These variation curves use the nodes marked out in Fig. 1a as abscissa to make a comparison among different leaders. Only two speed values can be calculated for the united leader I, and the last value is a lower bound because the leader stopped propagating when it reached the ground. It can be seen in Fig. 2c that the chased leader I did not show distinct differences from counterparts initially but then propagated at a relatively lower speed between Points C_2_ and D_2_. By contrast, the chasing leader I kept higher speeds all the way. The last speed of the chasing leader I was 9.75 × 10^6^ m/s based on the assumption that the leader head tip of it, in Frame 0, was at the position of the chased leader I’s tail in Frame -1. Although there were only two values calculated, the speed variation curve of the united leader I seems to indicate that the united leader I was the neutralization result of the chased leader I and the chasing leader I. The propagation speeds of the chased leader I ranged from 3.83 × 10^5^ m/s to 2.40 × 10^6^ m/s with the mean value of 1.10 × 10^6^ m/s, and those of the chasing leader I ranged from 5.66 × 10^5^ m/s to 9.75 × 10^6^ m/s with the mean value of 3.66 × 10^6^ m/s. The only two values of the united leader I were 3.88 × 10^6^ m/s and 3.70 × 10^6^ m/s, respectively.

To be concluded, the chased leader I and the chasing leader I propagated along the same path and met before reaching the ground. The chasing leader I entered the FOV of the high-speed camera 1.95 ms later than the chased leader I with a higher propagation speed. The propagation of the chasing leader I made the electric field signature increase. When the chasing leader I was close enough to the chased leader I, the chased leader I turned to propagate bidirectionally. After the two leaders met, the united leader I propagated at speeds in between those of the chased leader I and the chasing leader I, and then initiated the return stroke. Compared with counterparts, the duration of the leader-chasing behavior was much longer.

### Case II

**Figure 3:**
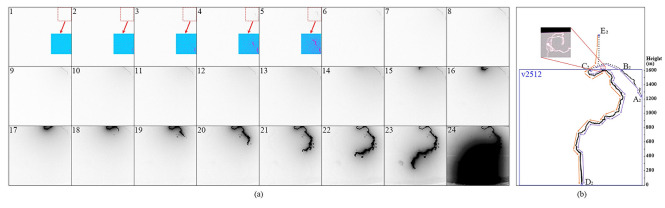
The illustration of the path along which the chasing leader II propagated and the speculative structure of the channel out of the high-speed camera’s FOV. (**a**) A sequence of cropped high-speed frames showing the propagation of Dart leader 8, with background removed, intensity inverted, and contrast enhanced. Some areas in Frames 1 to 5 were colored with contrast further enhanced. (**b**) A processed frame showing the path for the leaders. The blue rectangle indicates the high-speed camera’s FOV, the orange arrows indicate the path of the chased leader II, and the purple arrows indicate the path of the chasing leader II. A photo with higher image resolution recording the twisted portion of the channel is shown.

Case II was a classical triggered lightning flash with eleven return strokes, whose leader-chasing behavior occurred before the 4th return stroke. The chased leader II and the chasing leader II propagated along two paths sharing a common portion. The chasing leader II propagated along the same path with Dart leader 8 whose propagation is shown in Fig. 3a. In Fig. 3b, we mark out some nodes of paths for convenience. During the propagation of the chased leader II, Channel “A_2_-B_2_” was not illuminated, indicating that the initial propagation of the chased leader II was along another channel. In Fig. 3b, we draw one possible channel structure above the FOV (about 1600 m above the ground).

**Figure 4:**
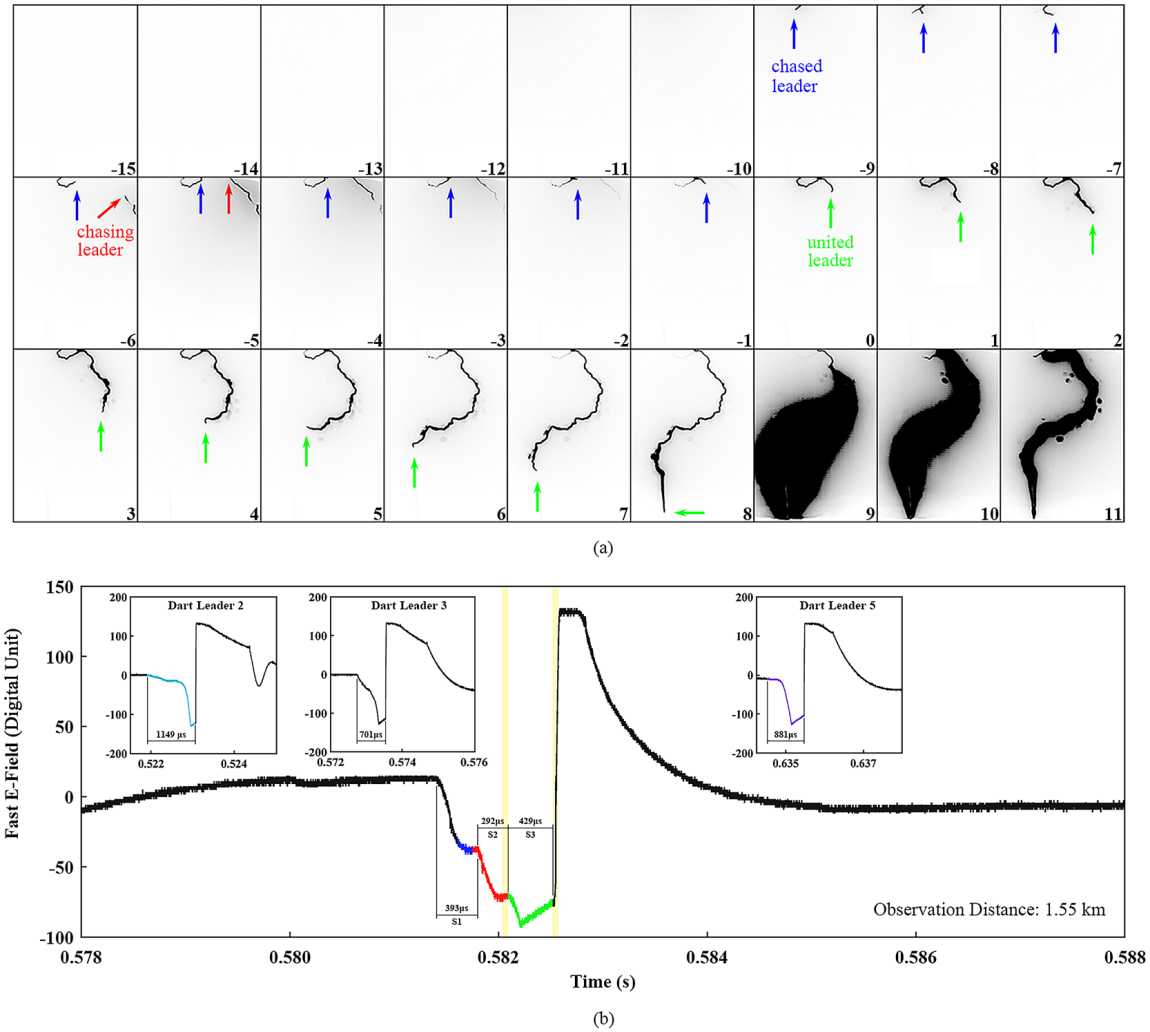
Optical recordings and corresponding electric field signatures of the leader-chasing behavior in Case II. (**a**) A sequence of cropped high-speed frames showing the leader-chasing behavior, with background removed, intensity inverted, and contrast enhanced. The arrows with different colors indicate the position of each leader’s head. (**b**) Corresponding electric field signature with three insets showing the counterparts.

Figure 4 illustrates the high-speed frames and corresponding electric field signatures of the leader-chasing behavior. It can be seen in Fig. 4a that Channel “A_2_-B_2_” was not illuminated, which indicates that the chased leader II may propagate along Channel “E_2_-C_2_”, before the chased leader II first entered the FOV. The chasing leader II occurred in Channel “A_2_-B_2_” in Frame − 6 and soon reached the upper boundary of the FOV in Frame − 5. From Frames − 4 to − 1, the head of the chasing leader II was out of the FOV while the tail of the chased leader was within the FOV. In Frame 0, the tail of the chased leader II seems to extend backward abruptly. This extension can be attributed to three possible reasons: (1) the chasing leader II died out or turned to propagate along a new path away from the FOV, and the chased leader II turned to be a bidirectional leader; (2) The chasing leader II continued its propagation and entered the FOV in Frame 0, and the extension was the head of the chasing leader II; (3) The chasing leader II entered the FOV in Frame 0 and the chased leader II turned to be a bidirectional leader, and the extension was the mixed chased leader II’s tail and chasing leader II’s head. Here we tend to attribute the extension to the head of the chasing leader II: firstly, the chasing leader II giving up propagating along its original path and the chased leader II turning into a bidirectional leader are less likely to occur at the same time; secondly, the tail of chased leader I began to extend backward about 150 μs earlier than when the two leaders met each other in Case I, which was longer than the possible duration (less than 50 μs) of the chased leader II’s backward extension. The height at which the two leaders met was over 1500 m, and the united leader II propagated continuously to initiate the 4th return stroke in Frame 9.

Figure 4b shows the electric field signature of this process, with three insets showing the three dart leaders which occurred within about 50 ms around the leader-chasing behavior. It should be noted that only the unsaturated portions of the electric field before return strokes were analyzed. We color the section corresponding to Frames − 9 to − 7 blue, Frames − 6 to 0 red, and Frames 1 to 8 green. The specific sections corresponding to Frames 0 and 9, where the two leaders met and the return stroke occurred, are also marked out with two yellow shadows. The electric field here is also divided into three segments: S1, S2, and S3. Dart leaders 2 and 3 propagated along the same path with the chased leader II while dart leader 5 propagated along the same path with the chasing leader II, which may cause difference in the electric field variation of Dart leader 5 and Dart leaders 2 or 3. The variations of S1 and S3 are similar to those of Dart leaders 2 and 3. Different from those counterparts, S2 has a similar decrease to that of S1, but has a shorter duration. The total duration of S1, S2, and S3 is 1114 μs, which is comparable with those of Dart leaders 2, 3, and 5.

**Figure 5:**
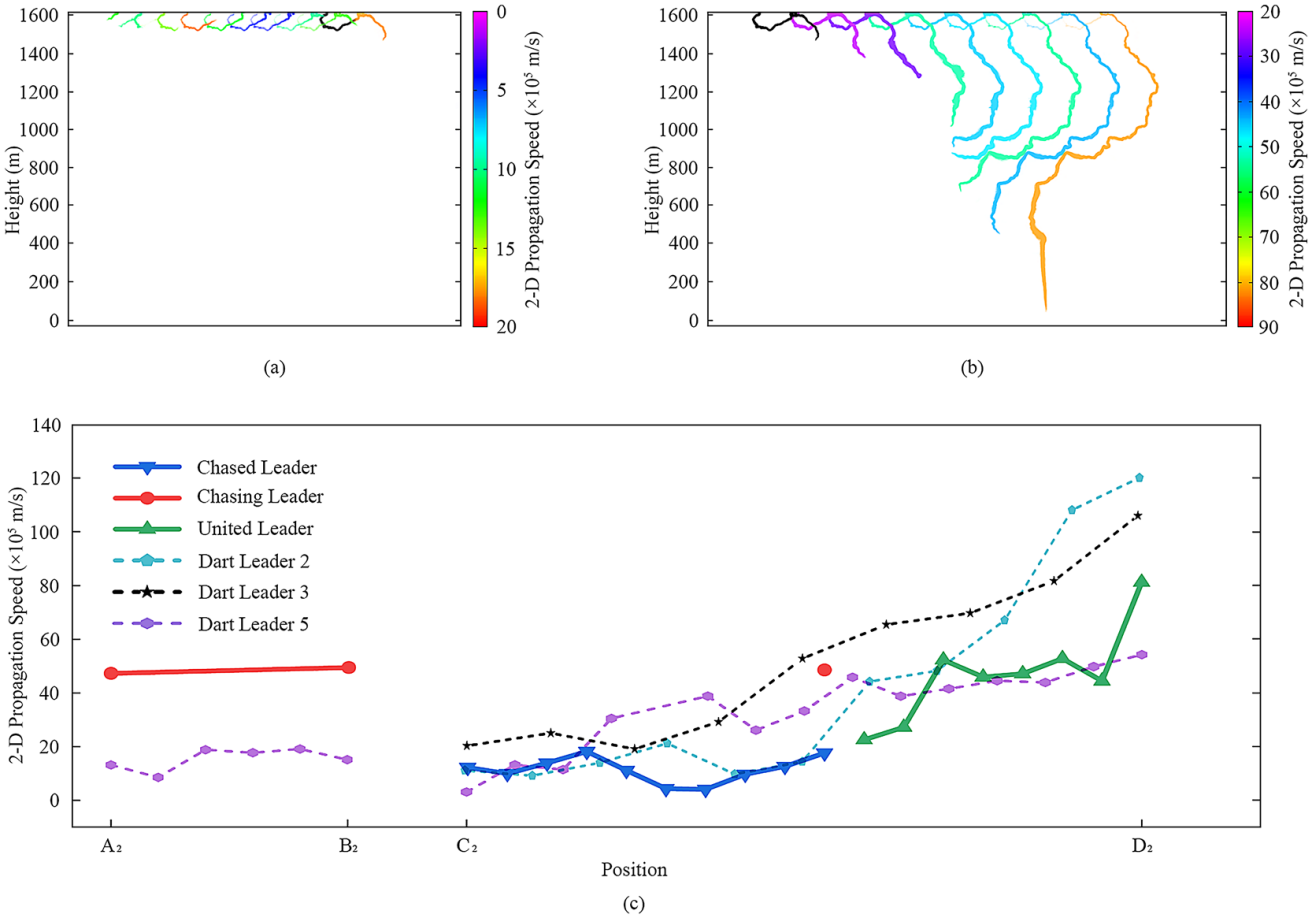
Propagation speeds of leaders in Case II. (**a**) The speed-position figure of the chased leader II. (**b**) The speed-position figure of the united leader II. (**c**) The propagation speeds variation of the chased leader II, chasing leader II, and united leader II, with three dart leaders as counterparts.

Figure 5 is drawn via the same method adopted for Fig. 2, illustrating the propagation speeds of leaders in Case II. Figure 5a reflects the propagation speeds of the chased leader II, and Fig. 5b reflects those of the united leader II. In Fig. 5a, we can find that the lower speed values occurred when the leader propagated in the twisted portion, which was shown in Fig. 3b. The lower speed values may be caused by the low image resolution of the high-speed camera, i.e. the exact position of the chased leader II’s head can’t be figured out when it extended in the twisted portion.

Figure 5c illustrates the propagation speeds of the chased leader II, chasing leader II, and united leader II, with three dart leaders mentioned before as counterparts. Only three speed values can be calculated for the chasing leader II, and the last value is a lower bound based on the assumption that the leader head (in Frame − 1) was at Point C_2_, and the leader head (in Frame 0) was at the position of the chased leader II’s tail in Frame − 1. It can be seen in Fig. 5c that the chased leader II and the united leader II did not show obvious differences from counterparts, while the chasing leader II had a relatively higher speed (considering the last value is a lower bound). Additionally, the variation curve of the united leader II seems to be well connected with that of the chased leader II, or, the development trend of the two curves is consistent. The propagation speeds of the chased leader II ranged from 4.13 × 10^5^ m/s to 1.82 × 10^6^ m/s with the mean value of 1.14 × 10^6^ m/s, and those of the united leader II ranged from 2.26 × 10^6^ m/s to 8.12 × 10^6^ m/s with the mean value of 4.67 × 10^6^ m/s. The first two speed values of the chasing leader II were 4.73 × 10^6^ m/s and 4.95 × 10^6^ m/s, respectively.

In conclusion, the chased leader II and the chasing leader II propagated along two paths sharing a common channel and met each other in the common channel before reaching the ground. The chasing leader II entered the FOV of the high-speed camera 150 μs later than the chased leader II with higher propagation speeds. The chasing leader II caught up with the chased leader II, making the luminous channel recorded in the frame longer. After the two leaders met, the united leader II propagated at speeds whose trend was similar to that of the chased leader II, and then initiated the return stroke. The duration of the leader-chasing behavior in Case II was comparable with those of counterparts.

## Discussion

As mentioned before, the propagation of a type *β*_2_ stepped leader is accompanied by one or more dart streamers or “luminous processes” that travel rapidly down along the previously formed channel to meet the leader tip of type *β*_2_ stepped leader^14–15^. Propagation speeds of most reported type *β*_2_ stepped leaders were not influenced distinctly by “luminous processes”. Although the propagation of type *β*_2_ stepped leader has a few differences from the leader-chasing behavior, it is to our knowledge the only reported phenomenon associated with the “chasing” behavior. Campos et al*.*^15^ reported that the speeds of “luminous processes” were between 10^6^ and 10^7^ m/s. They proposed that the “luminous process” is the visible manifestation of one or more recoil leaders. These recoil leaders begin inside the cloud and connect with another bidirectional leader, and then travel down to the lower end of the stepped leader path.

Although playing a similar role of being caught up, the chased leaders here are different from the type *β*_2_ stepped leaders, because they are dart leaders. They went downwards along the residual channel instead of virgin air, with a longer luminous body and higher propagation speeds. In addition, the chasing leaders are also not the same as the reported “luminous processes”. The chasing leaders here are more like leader processes than the “luminous processes”, with a clear propagation process. Most of the reported “luminous processes” in Campos et al.^15^ cropped up in only one or two frames and re-illuminated the channel left by the stepped leader, which was hard to recognize with little luminosity. The absence of the propagation process of “luminous processes” may be caused by the lower recording frame rate (4000 fps), the high speed of “luminous processes”, and the relatively smaller FOV.

The united leader could be a survived one between the chased leader and the chasing leader or a brand-new composite one. To figure out the property of the united leader, we need to combine the frames with the electric field signature. The electric field signature caused by the chasing leader I was quite different from that of the chased leader I. The signature was biased to the positive side to have an obvious increase lasting about 500 μs indicating that the chasing leader I is most likely a positive leader. The increased electric field signature is not caused by the propagation direction of the chasing leader I because the chased leader I propagated along the same path in the same direction while the electric field signature kept decreasing. The speed of the chased leader I was significantly influenced by the chasing leader I after they met, if we suppose the united leader I is the survived chased leader I. Additionally, the tail of the chased leader I extended backward as the chasing leader I was approaching, which is similar to the extension of the positive end of a space leader towards the negative leader tip as observed in laboratory sparks^21–26^. The increased electric field signature, influenced speed, and bidirectional development of the chased leader I make it clear that the chasing leader I is a positive dart leader. The united leader I is a negative composite leader, as indicated by the electric field signature and the speed variation. The positive chasing leader I significantly influenced the propagation of the chased leader I, which made the chasing leader I different from the description of those reported dart streamers or luminous processes.

The electrical field signature caused by the chasing leader II was similar to that of the chased leader II. At the same time, the speed variation of the chased leader II was not influenced distinctly by the propagation of the chasing leader II, if we suppose the united leader II is the survived chased leader II. And, the chased leader II did not show a clear bidirectional development like the chased leader I to meet the arrival of the chasing leader II. Therefore, we suggest that the chasing leader II is a negative recoil leader like the “luminous process” in type *β*_2_ stepped leader. Consequently, the united leader II is actually the chased leader II.

A critical question, which will be helpful in understanding the nature of the chased leader, is whether the chased leader will initiate a return stroke if the chasing leader does not catch up with it before reaching the ground or even does not exist. In Case II, when the chasing leader II appeared, the chased leader II was still an adequately intense leader with a long luminous body and relatively “normal” propagation speed. Moreover, the united leader II is believed to be the survived chased leader II. Therefore, the chased leader II will probably initiate a return stroke without the existence of the chasing leader II.

In Case I, we can find from Figs. 1b and 2a that the chased leader I was becoming weaker and weaker before the chasing leader I was close enough to it. Here we can predict three possible results of the chased leader I (if the chasing leader I was absent). The first result is that the chased leader I will continue to propagate and then initiate a return stroke. The second is that it will also initiate a return stroke but have its propagation paused before reaching the ground and then turn to be a bidirectional leader, just like the leaders reported by Qie et al*.*^27^. The last is that it will fail to reach the ground and turn to be an attempted leader. In the last situation, the chased leader I will play a role similar to that of the attempted leader reported by Lyu et al*.*^28^, with the chasing leader I being the subsequent dart leader inducing the return stroke.

In conclusion, the two cases reported here reflect two types of leader-chasing behavior. The first type occurs when two leaders with opposite polarities propagate in the same channel. In this type, the chasing leader will induce the chased leader to be a bidirectional leader when they are close enough. After they meet, a new composite leader will be formed and propagate at speeds in between those of the chased leader and the chasing leader. The return stroke will be initiated by the composite leader. The first type of leader-chasing behavior is quite different from the process occurring during the propagation of type *β*_2_ stepped leader. The occurrence of the chasing leader significantly influences the propagation of the chased leader and leads to the occurrence of the composite leader, while the occurrence of dart streamer hardly influences the propagation of type *β*_2_ stepped leader. The second type occurs when two leaders with the same polarity propagate in the same channel. In this type, the chasing leader will die out when it reaches the head of the chased leader, whose propagation does not result in obvious changes in the development of the chased leader. After the chasing leader dies out, the chased leader will keep propagating towards the ground and initiate the return stroke. This second type of leader-chasing behavior is similar to the process occurring during the propagation of type *β*_2_ stepped leader. The difference between these two processes is that the chasing leader is the dart streamer occurring during the propagation of a dart leader instead of a stepped leader. Observations on these two types of leader-chasing behavior help us to acquire a more intuitive understanding of the interactive processes between two different leaders with the same or opposite polarities and prove that return stroke can be initiated by a composite leader formed by a positive leader and a negative leader.

## Methods

The two flashes discussed here were triggered respectively on 2 and 7 July 2019 at the Guangzhou Field Experiment Site for Lightning Research and Testing in Conghua, Guangdong, China. On 7 July, the observations were made for an attempt at classical triggered lightning that resulted in an unintentional altitude trigger after the wire broke during the ascent period of the rocket. The flash was triggered, according to the recorded frames, just near the launch tower. The optical and electric field data presented here were obtained from an observation station positioned 1.55 km south of the launch tower.

The high-speed frames were recorded by a Phantom v2512 high-speed camera operating at a frame rate of 20 kfps, with an image resolution of 640 × 608 pixels (horizontal × vertical). The exposure time is 49 μs per frame (1 μs dead time), and the size of each pixel was 28 μm × 28 μm. The high-speed camera was coupled with a Nikon 16 mm lens at f/2.8 and located on the roof of the observation station. At a distance of 1.55 km, the spatial resolution was about 2.71 m per pixel. The still photo here was shot by a Canon 6D II camera coupled with a Sigma 12–24 mm lens at f/6.3. The focal length used here was 16 mm. The 2-D propagation speeds here were calculated between two successive frames based on the position of the leader head tip. The manufacturer of the high-speed cameras, Vision Research Inc., provides the operating software to control the equipment, record the data, and display the images. The electric field waveform was recorded by a multi-channel high-speed digital oscilloscope (DL850) with a sampling rate of 5 MS/s and a recording length of 2 s, with a time constant of 1 ms and a 3 dB bandwidth from 160 Hz to 1 MHz for the electric field sensor.
